# Single-atomic-ion detection with plasmon-enhanced whispering-gallery-mode microlasers

**DOI:** 10.1038/s41566-026-01882-7

**Published:** 2026-03-25

**Authors:** Samir Vartabi Kashanian, Frank Vollmer

**Affiliations:** 1 Living Systems Institute, University of Exeter, Exeter, UK; 2 Department of Physics and Astronomy, University of Exeter, Exeter, UK

**Keywords:** Microresonators, Imaging and sensing

## Abstract

Whispering-gallery-mode microlasers have emerged as powerful tools for label-free biosensing, yet their sensitivity has been limited to detecting nanoparticles larger than 10 nm. Here we demonstrate a plasmon-enhanced whispering-gallery-mode microlaser capable of detecting single atomic ions in solution, achieving unprecedented sensitivity. By integrating gold nanorods onto ytterbium-doped silica microspheres, we reduce the effective mode volume by approximately 1,000-fold and enhance the local electromagnetic field, amplifying the signal-to-noise ratio. The self-heterodyne detection of beatnote frequency shifts between split lasing modes enables the real-time monitoring of transient and permanent interactions of zinc (Zn^2+^) and cadmium (Cd^2+^) ions with nanorod sensing sites. We report peak sensitivities with beatnote shifts of 3.7 fm for Zn^2+^ and 7.2 fm for Cd^2+^, showcasing the potential of plasmon-enhanced whispering-gallery-mode microlasers for single-molecule and atomic-scale sensing applications, including in vivo probing.

## Abstract (web summary)

Plasmon-enhanced whispering-gallery-mode microlasers enable single-atomic-ion detection in solution. Real-time monitoring of transient and permanent interactions of zinc and cadmium ions as well as peak sensitivities with beatnote shifts of 3.7 fm for Zn^2+^ and 7.2 fm for Cd^2+^ are demonstrated.

## Main

Whispering-gallery-mode (WGM) microlasers have emerged as key platforms for nanoscale biosensing, offering bright, narrowband emission responsive to environmental changes^1^. These microlasers, using Raman^2–4^, quantum dot^5^, fluorescence-doped^6,7^ and rare-earth gain media^8,9^, are increasingly used in applications ranging from biosensing to optical barcoding^10^. Fabricated in diverse geometries (for example, spheres, toroids and capillaries), WGM microlasers often support low-threshold lasing in microstructures with a small footprint and modal volume, suitable for multiplexed and nanoscale sensing applications^11^.

State-of-the-art WGM microlasers such as microtoroids and glass microspheres detect nanoparticles as small as tens of nanometres, including dielectric nanobeads and viruses in air and aqueous media^12^. The most sensitive platforms use active WGM cavities based on erbium-doped silica or Raman gain^2,13^. Yet, despite advances in molecular detection including DNA hybridization^14^, refractive index changes in protein binding^15^ and protein–ligand interactions^16^, the single-molecule detection of individual chemical species remains elusive.

Although the Schawlow–Townes limit suggests that active WGM sensors could outperform passive ones in sensitivity^17^, practical implementations suffer from thermal noise, laser fluctuations and environmental instability in liquid-phase operation^2^. Additionally, experimental techniques for detecting frequency perturbations in microlasers caused by interactions with single molecules, amidst background noise in biosensing applications, remain challenging. These issues constrain the detection of extremely small WGM wavelength shifts associated with single-molecule interactions perturbing the cavity field.

Self-heterodyne detection of beatnote frequency shifts from WGM mode splitting, induced by nanoparticle-induced backscattering on microlasers, offers a promising approach for observing microlaser frequency shifts in sensing and to reduce noise^18,19^. Also, exceptional points of WGMs and WGM microlaser systems are explored as platforms that could enhance the sensitivity for detecting nanoparticles and biomolecules above their fundamental noise floor^20^. However, these systems have yet to reach the sensitivity required for single-molecule detection.

Plasmonic enhancement has enabled single-molecule detection in passive WGM resonators such as 100-µm-diameter glass microspheres via local field amplification by gold nanoparticles^21^. These passive WGM sensor systems detect molecules from binding-induced WGM shifts and linewidth changes through frequency scanning with external tunable lasers^22–24^.

Although plasmonic elements, such as gold nanorods (NRs), broaden the linewidth of WGM resonators and reduce their quality (*Q*) factors^25^, aligned NRs substantially enhance the near-field intensity^12,26,27^. This enhancement corresponds to a reduction in the effective mode volume by approximately three orders of magnitude. Consequently, the resonance shift response of the plasmon-enhanced (PE) cavity to single molecules is amplified, proportional to the intensity enhancement and the correspondingly reduced mode volume. This effect is the most pronounced at NR tips, enabling the detection of single molecules with molecular weights below 1 kDa and conformational changes in enzymes^12,28,29^.

In this study, we address the limitations of WGM microlasers by integrating plasmonic NRs onto WGM microsphere cavities with gain. This approach amplifies the frequency response of the lasing split modes, overcoming the challenges associated with detecting single molecules on microlasing sensing platforms.

By combining plasmonic near-field enhancement with the self-heterodyne read-out of lasing mode splitting, we develop a PE-WGM microlaser platform. This system elevates single-ion signals above the intrinsic noise floor, achieving femtometre-level resolution for detecting wavelength-shift perturbations caused by atomic ions and molecules in liquid-based sensing applications.

## Experimental design and sensing characteristics

### Experimental scheme

The resonators we used in this study were microspheres with diameters ranging from 70 to 110 μm, fabricated by melting the tips of optical fibres. Each microsphere was dip coated with two thin layers of Yb^3+^-doped sol–gel, similar to what is described in refs. ^30,31^. This coating process resulted in a doped silica layer approximately 0.5 μm thick, with a local rare-earth ion concentration of 10^19^−10^21^ ions cm^−3^, sufficient for achieving low-threshold and continuous-wave lasing. The resonators exhibited *Q* factors of 10^5^−10^6^ at the excitation wavelength band.

**Fig. 1:**
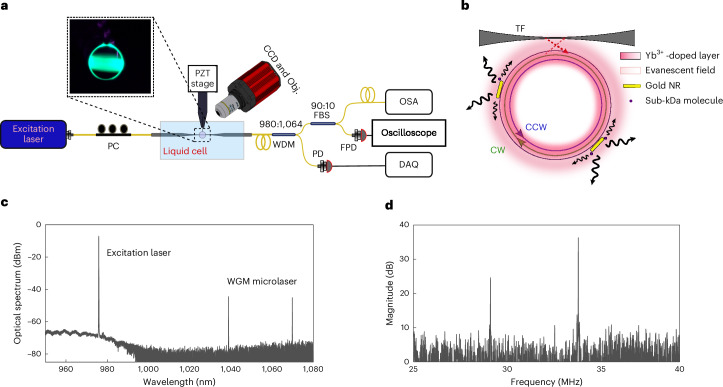
Experimental setup and microlaser mode splitting. **a**, Schematic of the experimental setup. CCD, charge-coupled device; Obj., objective; PC, polarization controller paddles; PZT, piezoelectric stage; WDM, wavelength-division multiplexer; FBS, fibre-based beamsplitter; FPD, fast photodetector; OSA, optical spectrum analyser; DAQ, data acquisition board. **b**, A PE-WGM microlaser is pumped by an external laser beam coupled via a TF, initially exciting the clockwise (CW) mode. The presence of plasmonic gold NRs—acting as plasmon enhancers for single-molecule detection—introduces scattering between the CW and counterclockwise (CCW) modes, resulting in the formation of SWMs with non-degenerate eigenfrequencies. This mode splitting gives rise to a beatnote signal in the laser output. When small molecules interact with the gold NRs, they disturb the splitting frequency of the SWMs, enabling sensitive molecular detection. **c**, Representative optical spectrum of a dual-mode microlaser, showing lasing peaks around 1,040 nm and 1,070 nm. **d**, Corresponding beatnote spectrum of the modes shown in **c**, with beat frequencies observed near 30 MHz and 34 MHz.

A 972-nm laser used to excite the gain medium via a tapered fibre (TF) coupled to the microlaser (Fig. 1), with emission typically observed between 1,030 nm and 1,100 nm. All the experiments were conducted in aqueous environments, and ytterbium was selected as it particularly provides high gain efficiency when the resonator is operated when immersed in water and emitting at a wavelength with excellent water transmission. Due to its high gain efficiency in water, sub-milliwatt excitation and lasing are enabled^13^.

Gold NRs were deposited by dipping the microresonator into 400 μl of HCl (pH 1.6), followed by injecting a small quantity of cetyltrimethylammonium bromide (CTAB)-capped NRs. This process ensured the stable adsorption of NRs onto the resonator surface, following the protocol in ref. ^25^. Although NR alignment was not controlled, their statistical distribution and occurrence of beatnotes ensured that at least one or more NRs are well positioned for local field enhancement, making single-molecule and atomic-ion sensing possible^25,32^.

The PE-WGM resonator was then coupled to the pump beam via a TF, which also collected the microlaser emission (Fig. 1). The system was immersed in aqueous media, such as a dilute caesium chloride (CsCl) solution, within a freshly prepared 500 μl of polydimethylsiloxane chamber. At the output, the pump and microlaser beams were separated using a wavelength-division multiplexer. A fast photodetector recorded beatnote oscillations in the microlaser emission, and their frequency components were extracted using fast Fourier transform processing on an oscilloscope at an acquisition rate of 22 Hz.

Although the oscilloscope’s limited acquisition rate constrained the temporal resolution, the fundamental resolution limit of the system is determined by the ultranarrow linewidth of the microlaser. In principle, time resolutions on the order of microseconds are achievable using a frequency discriminator, which converts frequency shifts into voltage signals recordable by high-speed acquisition systems^33^. Such fast detection is crucial for biosensing applications involving dynamic molecular events, such as enzymatic kinetics^34^.

### Beatnote sensing

**Fig. 2:**
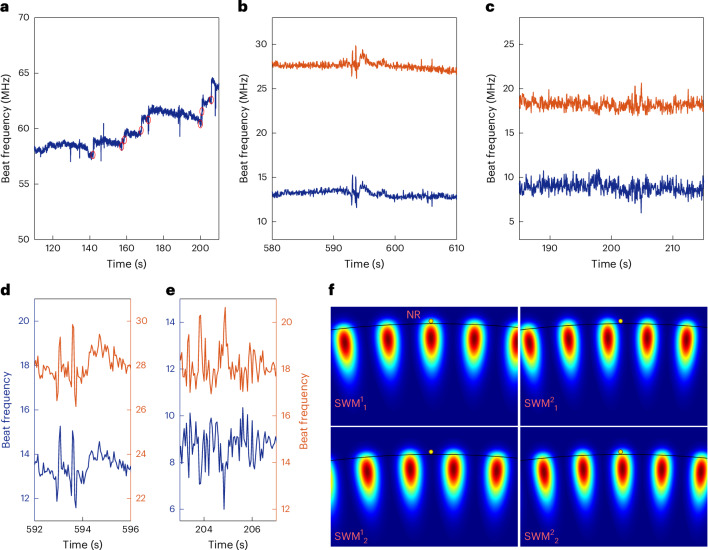
Sensing of single biomolecules and atomic ions. **a**, Detection of 50-nM GABA in water. The signal shows both permanent binding events—evident as step-like shifts—and transient interactions, which appear as sharp spike events. Step-like sensing events are highlighted with red circles. **b**, Transient spike signals corresponding to Zn^2+^-ion detection. **c**, Transient spike signals corresponding to Cd^2+^-ion detection. Dual lasing modes were used to monitor ion–NR interactions. **d**,**e**, Zoomed-in views of the sensing traces shown in **b** (**d**) and **c** (**e**), revealing individual spike events with higher temporal resolution. **f**, Simplified schematic illustrating how one gold NR contributes to a distinct sensing signal. In this specific example, the interaction of an analyte with this NR leads to a downshift on beatnote 1 and upshift on beatnote 2, similar to what is demonstrated in **e**.

To read out the frequency shift induced by single molecules interacting with the PE microlaser sensors (Fig. 2), we make use of the mode-splitting phenomenon induced by the interaction of plasmonic nanoparticles with the microlaser. This effect facilitates the self-heterodyne detection of microlaser frequency shifts that are too subtle to be resolved by a conventional spectrometer.

Each WGM consists of two degenerate counterpropagating travelling modes. A WGM microlaser emits in both degenerate modes, and their evanescent fields probe the surrounding environment. However, when a plasmonic scatterer interacts with the microlaser’s evanescent field, the degenerate lasing modes couple through intracavity Rayleigh backscattering, forming two non-degenerate standing-wave modes (SWMs), denoted here as SWM_1_ and SWM_2_ (refs. ^13,35,36^). The splitting between these two SWMs depends on the polarizability and nanoparticle position within the mode volume^36^. Binding of any new nanoparticle is, therefore, translated into a change in the splitting of the two SWMs^37,38^. Likewise, any variation in the polarizability near the existing nanoparticle alters the splitting, including the effective excess polarizability induced by the binding of a molecule at the tip of a gold NR.

The evanescent field of the WGM resonantly excites the localized surface plasmon of the NR, effectively coupling the microresonator with the sensing sites on the NR’s surface. These sensing sites correspond to localized intensity hotspots, predominantly located at the tips of the NRs. When a molecule or atomic ion enters these sensitive sites and interacts with the NR (Fig. 1b), the coupling between the NR and the microcavity converts this interaction into a redshift (Δ*λ*) of the WGM’s resonance position^12,39^. The shift, however, will be of a different magnitude for the two non-degenerate modes, which leads to a variation in their difference frequency and in the splitting frequency (Δ*ω*); this can become larger or smaller on molecules binding to the NRs^40^.

If the interaction is permanent, such as the formation of a strong covalent bond, a shift in the beat frequency, Δ*ω*, remains unchanged and it is manifested as a step-like signal in the beat frequency. However, if a molecule or an atom interacts transiently, remaining within the sensing site only for a limited time *τ*, the splitting frequency shifts back to its original state once the molecule departs. Therefore, tracking the beatnote frequency in real time probes the single molecules and even atom ions reported here, permanently (step signals) or transiently (spike signals) interacting with the sensing cites within the time resolution of the sensor set by the update rate of the oscilloscope.

### Laser mode splitting

The microlasers exhibited multiwavelength emission, with 1–4 lasing modes per resonator. The degree of mode splitting depends on the spatial overlap with the deposited NRs^13^, resulting in multiple fast Fourier transform peaks (Fig. 1c,d). Different lasing modes respond uniquely to each sensing event, which can enhance statistical confidence and reduce false detections.

Molecule-induced laser mode splitting in PE-WGM microlasers varies in sign and magnitude, typically following a log-normal distribution^41^. However, the orientation and chemical groups and function of the molecule interacting with the gold NR surface are not present in ions and, therefore, do not affect the ion–NR interactions. As a result, the sensing signal arises dominantly as a function of the near-field intensity near the NRs. This leads to a nearly Gaussian distribution, which agrees with our observation. In this picture, ions probe the near-field distribution, whereas molecules are more complicated and oversample/undersample certain locations and intensities due to multiple factors including their functional groups^42^. Variability in ion sensing arises from the following. (1) Binding site locations: hotspots (for example, nanoparticle tips) yield larger splitting than low-field areas. (2) Number of active nanoparticles: several gold NRs interact with the WGM field. Other factors include NR orientation, positional heterogeneity (equatorial versus polar) and cavity asymmetries, causing a range of coupling strengths and broadened mode-split histograms and statistics.

### Noise characterization

Thermal fluctuations shift both split modes of the PE-WGM microlaser in the same direction, but thermorefractive noise, which reduces mode coherence, is mitigated in split-mode sensing^43^. Other environmental factors, such as mechanical instability in the TF resonator gap or pump laser detuning, may induce slow frequency drifts. For instance, we found that the fine-tuning of the pump laser’s detuning could slightly modify the splitting (Supplementary Fig. 10). In our experiment, the excitation laser’s frequency is naturally stabilized, owing to the thermo-optical properties of the WGMs^44^, maintaining the pump laser–WGM detuning and alleviating potential slow drifts and shifts in the detuning. Beat frequency fluctuations under thermal locking and in the background CsCl buffer (5 mM) did not exceed ±100 kHz (Supplementary Figs. 7 and 8). Each noise analysis was conducted over a 10-min interval, using the same lasing modes used in the corresponding sensing experiments.

## Sensing single molecules and atoms

### Detecting GABA

Single-molecule detection was demonstrated using a 90-μm PE microlaser exposed to 50 nM of γ-aminobutyric acid (GABA) in water at pH 7.1. Emission at 1,035 nm produced a beatnote with a signal-to-background ratio of ~14 dB.

GABA is zwitterionic at pH 7.1, possessing both carboxylate (–COO^−^) and ($-{\mathrm{NH}}_{3}^{+}$) groups of opposite polarity, each interacting differently with a charged NR^41^. One group gives rise to transient events, whereas the other produces more permanent binding depending on the charge at the gold NR surface. The surface charges relevant to the experiment are unlikely to be those of the CTAB bilayer (which does not coat the NR tips; Methods), but rather those present at the tips themselves—negative charges originating from the gold surface and from the adsorbed carboxylate or carbonate species present in the aqueous buffer through the dissolution of atmospheric CO_2_.

### Detecting Zn^2+^

**Fig. 3:**
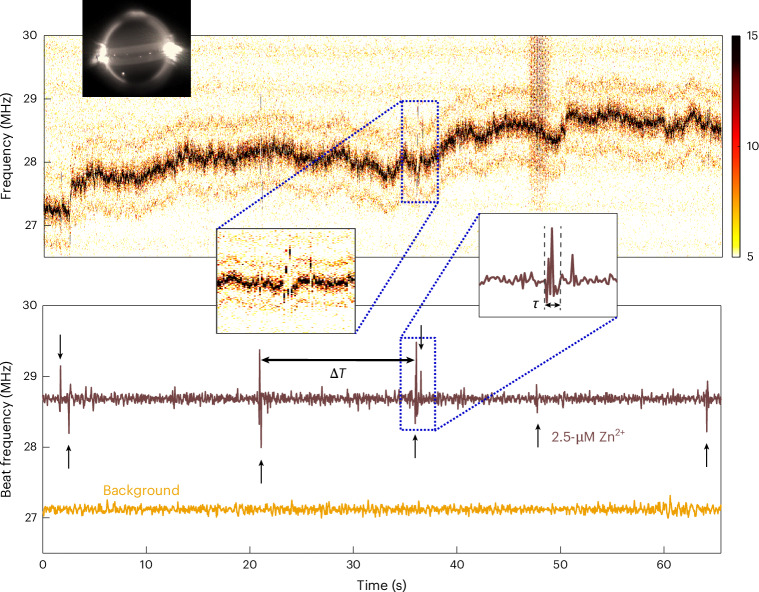
Transient ion–NR interactions and spike pattern analysis. Top: raw noise spectra of microlaser mode splitting under ion interaction, represented as a colour map compiled from a stack of 1,410 beatnote spectra. This visualization highlights the temporal variation in the beat frequency. Bottom: extracted and detrended beat frequency signal (brown line), corresponding to sensing events from a 2.5-μM Zn^2+^ solution. The intrinsic sensor noise is shown in yellow for comparison. Δ*T* denotes the waiting time between successive spike events, and *τ* represents the duration (dwell time) of an individual spike. Insets display two representative spike events as captured in both raw and processed data. The image of the PE-WGM microlaser used in this experiment is shown. The scattering of the microlaser from some of the deposited gold NRs is visible in the image as bright scattering spots.

**Fig. 4:**
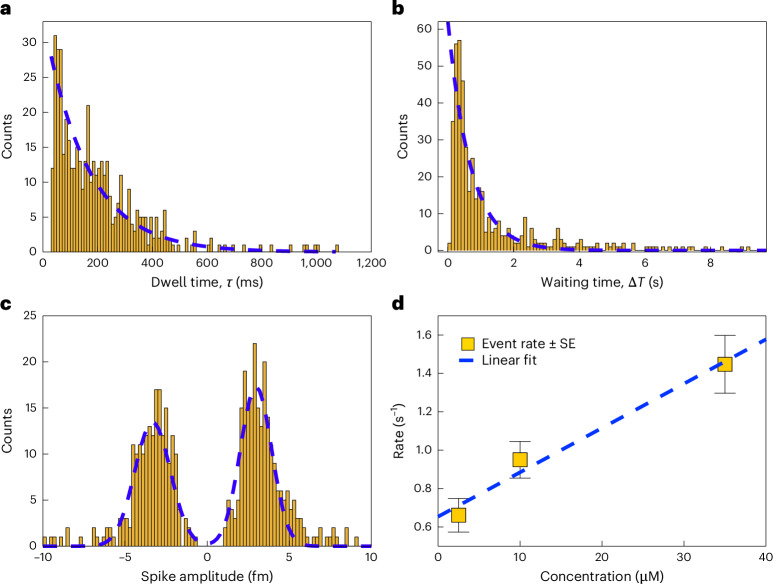
Statistical analysis of spike signals from Zn^2+^–NR interactions. A total of 461 spike events were recorded from a 35-μM Zn^2+^ solution in the presence of 5-mM CsCl. The background noise level (peak to peak) was measured at 1.4 fm; spikes with amplitudes below this threshold were excluded from the analysis to avoid false positives. **a**, Histogram of spike durations (dwell times, *τ*) in milliseconds. The dashed blue line represents an exponential fit to the data, yielding a characteristic time constant of 169 ± 19 ms. **b**, Histogram of waiting times between consecutive spikes, demonstrating a Poisson-like distribution. An exponential fit (dashed line) gives a spike rate of 1.5 ± 0.2 s^−1^. **c**, Histogram of spike amplitudes, along with a double Gaussian fit (dashed blue line), showing a mean magnitude of 3.7 fm and a standard deviation of 1.4 fm. **d**, Spike rate as a function of Zn^2+^ concentration, revealing a linear response over the measured range. These rates and their standard errors (SE) were determined by fitting an exponential decay function to waiting-time histograms containing 131, 400 and 461 events at 2.5, 10 and 35 μM of Zn^2+^, respectively.

As a benchmark, we tested transition metal ions with filled *d* orbitals, including Zn^2+^ and Cd^2+^. CsCl (5 mM) acted as a charge-screening agent, establishing a Debye length of *λ*_D_ ≈ 4.3 nm at 25 ^∘^C (ref. ^45^). This setup promoted weak NR–ion interactions, appearing as beatnote frequency spikes (Fig. 3). Using a 90-μm microlaser emitting at 1,030 nm and 1,033 nm, we first recorded baseline beatnote fluctuations in CsCl. The mean local standard deviation over a span of 3 s was ~90 kHz, with peak-to-peak variations below 400 kHz—equivalent to ~0.3–1.4 fm in wavelength. Zn^2+^ was introduced at concentrations of 2.5, 10 and 35 μM. For each condition, three datasets were recorded over 15 min. Beatnote shifts occurred in both directions, depending on the interaction with SWM_1_ or SWM_2_. Most interactions were transient (spike events), although step-like events were also observed: 20%, 7% and 5% of total events at 2.5, 10 and 35 μM of Zn^2+^, respectively. This decreasing trend suggests the saturation of permanent binding sites. The average spike amplitude was ~3.7 fm. The dwell time (*τ*)—the duration of each spike—provides insights into the interaction dynamics^39^. The waiting time (Δ*T*) between the spikes followed a Poisson distribution and decreased linearly with increasing Zn^2+^ concentration (Fig. 4), consistent with single-atom sensing behaviour. Spikes were recorded at the rates of 0.661 ± 0.088, 0.95 ± 0.096 and 1.5 ± 0.2 events per second.

### Detecting Cd^2+^

**Fig. 5:**
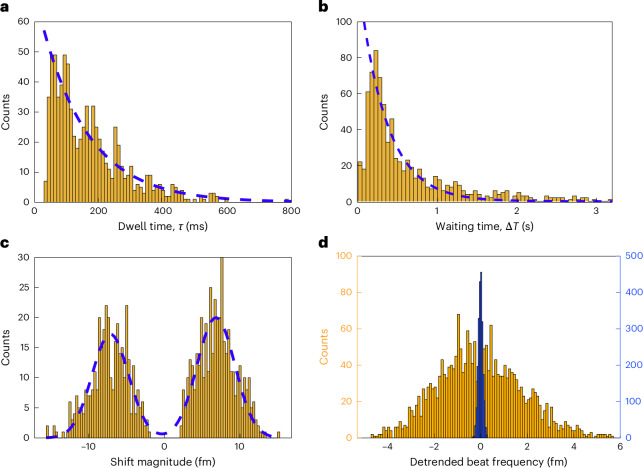
Statistical analysis of spike signals from Cd^2+^–NR interactions. A total of 786 spike events were recorded from a 5-μM Cd^2+^ solution in the presence of 5-mM CsCl. The background noise level was measured at 0.4 fm. **a**, Histogram of spike durations (dwell times, *τ*), showing an exponential decay with a time constant of 149 ± 16 ms. **b**, Histogram of waiting times between consecutive spikes. An exponential fit (dashed blue line) yields a spike rate of 3.8 ± 0.4 s^−1^. **c**, Histogram of spike amplitudes with a double Gaussian fit (dashed line), indicating a mean magnitude of 7.2 fm and a standard deviation of 3.5 fm. **d**, Histogram of the detrended beat frequency signal, centred at zero. The blue histogram represents the background noise, whereas the gold histogram corresponds to the sensing signal. For additional analysis, see appendix E in the Supplementary Information.

For cadmium detection, an 85-μm microlaser was used with four emission modes between 1,050 and 1,060 nm. Two beatnote signals with a signal-to-background ratio of >10 dB were selected. Beatnote peak-to-peak noise remained within ±100 kHz (~0.4 fm). A 5-μM CdCl_2_ solution in 5-mM CsCl buffer yielded a spike rate of 3.8 ± 0.4 events per second, with an average beat shift of 7.2 fm (Fig. 5). Only two step-like events were observed over 20 min of measurement time. The higher-amplitude shifts compared with Zn^2+^ align with cadmium’s higher polarizability^46^.

The magnitude of a sensing event strongly depends on the local near-field intensity at the sensing site for each SWM. It is important to note that molecular interactions always induce a redshift in each individual SWM. However, the relative magnitudes of the shifts in SWM_1_ and SWM_2_ determine the direction of shift in mode splitting. A greater redshift in SWM_1_ compared with SWM_2_ results in a decrease in the beatnote frequency (that is, reduced splitting when SWM_2_ > SWM_1_), and vice versa (Fig. 2d,e).

**Fig. 6:**
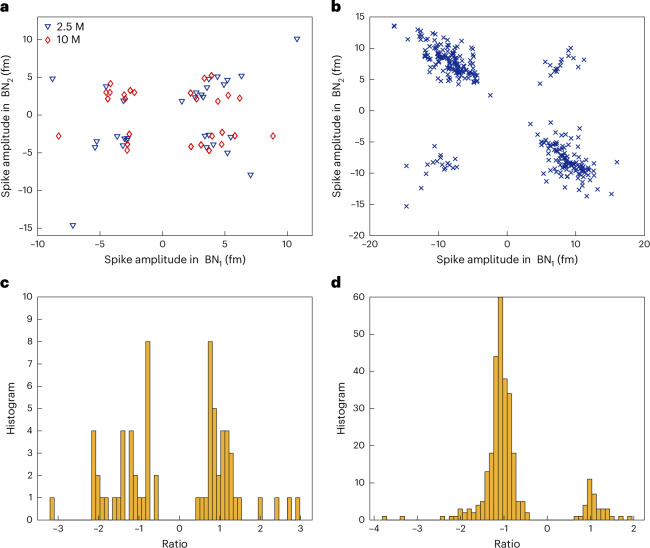
Coincident spike amplitudes in dual-beatnote signals. **a**, Coincident spike amplitudes observed in two distinct beatnote signals (BN_1_ and BN_2_) during zinc sensing at concentrations of 2.5 μM and 10 μM. **b**, Corresponding spike amplitudes from cadmium-ion detection at 5 μM. **c**,**d**, Histograms of the amplitude ratios between coincident detections in BN_1_ and BN_2_ for zinc and cadmium experiments, respectively. The distribution in **c** suggests that zinc sensing involves at least two NRs contributing comparably to the signal. By contrast, the narrower distribution in **d** indicates that cadmium sensing is predominantly mediated by a single NR, with at least one additional NR contributing at a lower detection rate.

In the case where the same sensing site is interacting with multiple lasing modes, the corresponding shifts in the split SWMs associated with each lasing mode *j* depends on the spatial field distribution of the corresponding SWMs, denoted as ${\mathrm{SWM}}_{i}^{j}$, where *j* indexes the lasing mode. For a given sensing site, this implies that simultaneous detections across different lasing modes will exhibit shifts with a preserved ratio in magnitude and relative direction. Specifically, the beatnote responses for a single sensing site may appear as up/up, up/down or down/down combinations; however, the relative pattern remains consistent for that site (Fig. 2f). Therefore, the ratio between coincident sensing spikes can be used as a proxy for estimating the number of gold NRs involved in the sensing^47^. Our analysis on dual-beatnote sensing signals aligns with the prediction of this theory (Fig. 6).

Importantly, for a different lasing mode, the field distribution at the positions of NRs may be inverted, resulting in opposite beatnote shifts at the same sensing sites. Our experimental data support this behaviour, demonstrating that a perturbation at a single sensing site can generate beatnote shifts of either the same or opposite direction across multiple modes (Fig. 2d,e). We also observe that beatnote spikes are sometimes followed by a subsequent backaction, which we attribute to the intrinsic dynamics of the microlasing process, probably associated with the relaxation behaviour arising from pump laser–WGM detuning (Supplementary Fig. 10).

## Conclusion

The frequency shift observed in split WGMs on interaction with a particle or molecule is governed by the excess polarizability of the analyte relative to the surrounding medium, weighted by the local light intensity at the site of interaction^48^. Enhancing the optical field at the analyte’s location can, thus, amplify the measurable shift. Such a field enhancement typically occurs at the surfaces of plasmonic nanoparticles when excited near their resonance^49^. For small analyte molecules (molecular weight of <8 kDa), this localized field amplification is the dominant mechanism underlying the observed resonance and split-mode shifts. However, relying solely on the polarizability of single ions to induce detectable WGM shifts requires additional local enhancement factors on the order of ~1,000, to match the spectral shifts observed with those predicted by cavity perturbation theory^25,41^. Moreover, we did not observe any increase in noise attributable to background ions from the buffer solution, suggesting that neither the polarizability nor the charge of individual ions alone is sufficient to explain the observed splitting shifts^50^, and indicating that surface chemistry plays a role in determining which ions and their interaction kinetics can be detected by the sensor.

We propose that additional mechanisms, potentially linked to atomic-scale field enhancements arising from the intrinsic surface roughness of gold NRs, contribute significantly to the observed effects. This interpretation is supported by previous reports of strong coupling between single molecules and plasmonic picocavities at room temperature^51,52^.

Looking ahead, the reduction in mode volume provided by plasmonic enhancement implies a stronger Purcell effect, which could enable a coherent coupling to two-level systems, as previously demonstrated in ref. ^53^. Additionally, the sensitivity of the system opens up opportunities to detect dynamic processes, such as photoacoustic oscillations, through sideband transduction in the microlaser’s emission spectrum^54–56^, potentially extending to the detection of single proteins^57,58^. These capabilities open exciting possibilities for transducing complex molecular dynamics in real time with unparalleled sensitivity.

A particularly promising direction involves leveraging PE-WGM microlasers for in vivo applications. For example, the laser beatnote could be extracted from microbeads embedded in living organisms, using prism-based or total internal reflection objectives to couple the signal into a single-mode waveguide for real-time beatnote analysis. Such a platform could ultimately support single-molecule sensing in biological environments, pending the development of robust far-field coupling techniques.

In particular, WGM microlasers with linewidths at the hertz level have already been demonstrated using similar doping strategies^59^, suggesting that ultranarrow linewidths—and thus improved detection limits—are attainable. In our current experiments, however, the detection limit was primarily constrained by the measurement instrumentation and the lack of active stabilization of the pump laser. Incorporating a stabilization module such as a Pound–Drever–Hall lock-in scheme could significantly improve system stability.

Moreover, our study used a standard Yb doping process, but further refinement—such as improving dopant uniformity and avoiding clustering of Yb ions—could enhance the consistency and performance of the microlasers. In addition mechanical vibrations and coupling instability associated with TF coupling may introduce noise; transitioning to integrated on-chip microring lasers, for instance, could mitigate these effects and further improve the detection thresholds. Additionally, implementing real-time self-referenced linewidth monitoring, as described in refs. ^60,61^, could provide a robust mechanism for tracking spectral changes with high precision, which ultimately could allow single-shot optical mass spectroscopy.

In summary, and to the best of our knowledge, this work presents the first demonstration of single-atomic-ion detection using PE-WGM microlasers, leveraging the self-heterodyne detection of split-mode frequency shifts. This platform paves the way for ultrahigh-sensitivity sensing of atomic-scale analytes in aqueous environments.

## Methods

### Fabrication of Yb^3+^-doped WGM microlasers

Yb^3+^-doped WGM microlasers were used in all the experiments. The fabrication procedure began with the formation of microspheres by melting the tips of single-mode fused silica optical fibres (SMF-28) using a CO_2_ laser^22^. Once the desired microsphere size was achieved, a sol–gel solution doped with Yb^3+^ ions was prepared^31^. The microspheres were then dip coated in the sol–gel solution for 30 min and baked at 150 °C for another 30 min to consolidate the coating. This dip-coating and baking processes were repeated twice, resulting in an ~0.5-μm-thick doped silica layer on each resonator. The final coating thickness was determined by the sol–gel’s viscosity, the duration of dip coating and the number of coating cycles. Over time, the sol–gel solution naturally thickens and eventually solidifies, limiting its usable lifetime. Following the dip-coating steps, the doped microspheres were reflowed by a CO_2_ laser to melt and fuse the doped silica layer uniformly into the surface of the microspheres.

### Chemical preparation and buffer selection

All chemicals were purchased from Sigma-Aldrich and freshly prepared before each experiment. CsCl was used as a charge-screening agent. CsCl was chosen over more conventional salts such as sodium chloride due to its superior compatibility with the sol–gel layer. Sodium ions are known to disrupt Si–O–Si bonds, creating non-bridging oxygen sites^62^, which we found to degrade beatnote stability and negatively affect lasing efficiency. Caesium ions, being larger and less mobile, produced less interference and did not degrade microlaser performance. All solutions were prepared in MilliQ water and filtered using 0.2-μm membrane filters (Sartorius Minisart).

### Plasmonic NR integration

To achieve plasmonic enhancement, we used CTAB-capped gold NRs from NanoPartz (A12-10-1064-CTAB-DIH-1-25), with a length of 67 nm, diameter of 10 nm and a plasmon resonance centred at 1,060 nm. The NRs were attached to the microlaser resonators in a separate chamber to avoid unintended deposition on the TF. A custom-fabricated 500 μl of polydimethylsiloxane liquid chamber was used for all the experiments. The PE microlaser and TF were immersed in a 5-mM CsCl solution and observed using a ×10 objective lens and a camera system.

### Optical characterization and laser excitation

The *Q* factor of each resonator at the pump wavelength (~972 nm) was measured by scanning the laser across a resonance and fitting the transmission spectrum to a Lorentzian function. The *Q* factors of the resonators ranged from 2 × 10^5^ to 5 × 10^5^. For lasing, the pump laser was slightly blue-detuned from the WGM resonance. This slight detuning enabled passive frequency locking to the WGM, which maintained stable lasing for tens of minutes without active stabilization^44,63^. This locking behaviour ensured sufficient data acquisition time without mode drift or loss.

### Beatnote signal characterization

Before the sensing experiments, the lasing modes were characterized to assess their stability and signal quality. Only beatnote signals with a signal-to-background ratio exceeding 10 dB were considered valid for the sensing analysis. This criterion corresponds to beatnote peaks with magnitudes more than 10 dB above the background fluctuation level in the absence of beatnote peaks.

## Online content

Any methods, additional references, Nature Portfolio reporting summaries, source data, extended data, supplementary information, acknowledgements, peer review information; details of author contributions and competing interests; and statements of data and code availability are available at 10.1038/s41566-026-01882-7.

## Supplementary information

Supplementary Information Supplementary Figs. 1–14, Appendices A–K and Tables 1 and 2.

## Data Availability

The data used in this work are available from the corresponding authors upon reasonable request.
