# Supplementary material for: Single-atomic-ion detection with plasmon-enhanced whispering-gallery-mode microlasers
Source: Nat Photonics. 2026 Mar 25;20(4):404–12. doi: 10.1038/s41566-026-01882-7 (PMC13061644; doi:10.1038/s41566-026-01882-7)
Supplement: Supplementary file 1 — Supplementary Figs. 1–14, Appendices A–K and Tables 1 and 2. [file 41566_2026_1882_MOESM1_ESM.pdf]

nature photonics

Supplementary information

https://doi.org/10.1038/s41566-026-01882-7

Single-atomic-ion detection with plasmon-enhanced whispering-gallery-mode microlasers

In the format provided by the authors and unedited

Appendix A: Sol-gel Preparation and Microlaser Fabrication

The sol-gel was prepared following the protocol described in [1]. A mixture of 7.395 mL tetraethyl orthosilicate (TEOS), 1.77 mL water, and 1.901 mL ethanol was prepared. To initiate the reaction, 0.66 mL of 0.1 M HCl was added, and the mixture was vigorously stirred at 70 °C for 5 minutes. The initially opaque solution turned transparent after four minutes of stirring. Next, 227.1 mg of $Yb(NO_3)_3.5H_2O$ (99.9%, Thermo Fisher Scientific) dissolved in 1.465 mL ethanol was added. The doped sol-gel was aged for 4.5 hours at room temperature.

Microspheres ($70 - 110\,\mu$m in diameter), fabricated by melting the tip of a single-mode fiber (SMF-28) using a CO_2 laser, were dip-coated in the sol-gel for 30 minutes. The coated spheres were then baked at 150° C for 30 minutes. We repeat the coating process one more time. Each coating add a few 100s nm thick layer of the sol-gel to the surface, depending on the viscosity of the sol-gel and the coating duration [2]. Finally, the coated spheres were reflowed using a CO_2 laser to create a smooth, uniform sol-gel layer fused to the silica surface.

Appendix B: Theoretical expectation from the Schawlow–Townes limit

Using typical parameters of our Yb microlasers ($\lambda \approx 1060$ nm, cold-cavity Q$\approx 10^5 - 10^6$, $P_{out} \approx 1\,\mu$W), the fundamental Schawlow–Townes linewidth is estimated to be $\Delta\nu_{ST} = (\pi h\nu^3)/(P_{out}Q^2) \approx 50$ kHz–5 MHz. This corresponds to an effective lasing Q-factor of $\approx 5 \times 10^7$ to 5×10^9, i.e., a linewidth narrowing (and hence Q enhancement) of up to $\sim 10^4$ relative to the cold cavity, consistent with earlier reports of strong linewidth suppression in active WGM microcavities [3].

Appendix C: Chemicals Preparation

The analytes used in the sensing experiments were 99% γ-aminobutyric acid (GABA), 99.999% $ZnCl_2$, and 99.99% $CdCl_2$, all from Sigma-Aldrich. Cesium chloride (CsCl, 99.9%, Sigma-Aldrich) was added at 5 mM concentration to screen charge near the nanorod tips. All solutions were prepared in Milli-Q ultrapure water and filtered through a $0.2\,\mu$m membrane (Sartorius Minisart). Plasmonic enhancement was achieved using gold nanorods (A12-10-1064-CTAB-DIH-1-25, NanoPartz Inc.) with 67 nm length, 10 nm diameter, and resonance at 1060 nm.

Appendix D: Single ions vs clustering

Confirmation that the observed transient mode-splitting events arise from single ions rather than small ion clusters rests on the following arguments:

1. **Solution chemistry and speciation** All experiments were performed in ultrapure water at near neutral pH ≈ 6.5 with non-coordinating counterions (nitrate or perchlorate). Under these conditions and at the low analyte concentrations used (a few μM), both Zn^{2+} and Cd^{2+} exist almost exclusively as fully hydrated monomeric $[M(H_2O)_6]^{2+}$ species.
   - For Cd^{2+}, hydrolysis and oligomerisation are negligible below pH 7 and at μM concentrations; the predominant form is the monomer (> 99.9%) [4].
   - For Zn^{2+}, very weak and reversible dimerisation or hydrolysis may begin at higher concentrations or pH, but thermodynamic data and speciation modeling (Visual MINTEQ, PHREEQC) predict < 0.5% of Zn present as dimers or hydroxo-bridged species under our exact conditions.
2. **Signal amplitude distribution** Species with twice the polarisability (e.g. molecular dimers) lead to WGM resonance shifts roughly twice the amplitude compared to those observed for monomers. We have previously resolved such populations, for example in glutamate dimer measurements [5], confirming that double-amplitude peaks for glutamate-glutamate are detectable with our approach; it is known that glutamate can dimerise [6, 7]. No such secondary population is present in either Zn^{2+} or Cd^{2+} amplitude histograms, and also not seen for GABA.
3. **Concentration dependence** The event rate scales strictly linearly with concentration over more than one decade. If clusters formed at the sensing surface, a quadratic (or higher-order) dependence would be expected; this is not observed.

Taken together, these chemical, statistical, and kinetic observations provide strong evidence that the great majority of detected transient events correspond to single Zn^{2+} or Cd^{2+} ions rather than clusters.

Appendix E: Data Acquisition

Beatnote signals were detected using a 5 GHz bandwidth fast photodetector (DET08C, Thorlabs) and amplified by +25 dB using a broadband amplifier (ZFL-500-BNC, Mini-Circuits). The signal was recorded using a PicoScope 5444B oscilloscope. Fast Fourier Transforms (FFTs) were used to extract beatnote frequencies. The typical frame rate was ∼40 ms, which defined the time resolution of our measurements.

Time resolution can be improved to sub-millisecond levels using a real-time spectrum analyzer or a custom RF discriminator that converts beatnote frequency into analog voltage, as described in [8, 9]. Such improvements could be valuable for probing dynamic biomolecular processes, such as protein conformational changes.

Appendix F: Identifying Sensing Events

The recorded spectra then is read and a surface plot is generated as a useful visualization technique. This is done by stacking a group of spectra in a 2D area. Then a Matlab code, which is based on the Matlab built-in peak finder function, is used to register the frequency and amplitude of the beatnotes in the spectra (Figure 1, 2). The extracted time-trace beat frequency then is used in our analysis.

The schematic in Fig. 3 demonstrates the signal-processing pipeline applied to the raw beatnote data, yielding a detrended time trace suitable for spike analysis. This analysis quantifies the timing, duration, direction, and amplitude of sensing events. To detrend the signals and eliminate slow drifts and variations, we first applied a Savitzky–Golay filter to identify potential detection spikes. Regions where signal variations exceeded a predefined threshold were excluded from the filtering process. Interpolation was then used to reconstruct the excluded segments, generating a complete trend line. Subtracting this filtered trace from the original signal removed the slow drifts, as shown in Figs. 4 and 5. This approach closely follows the method described in the supplementary information of [10].

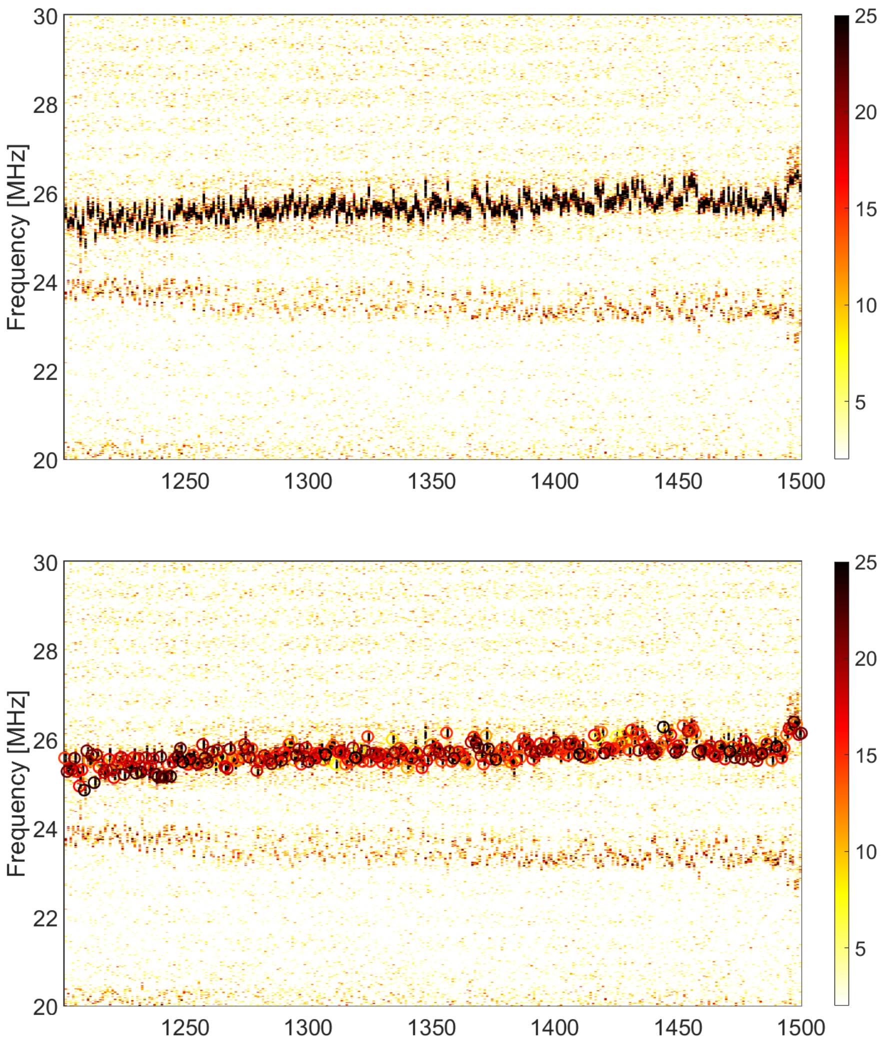

FIG. 1. Extraction of beat frequency from raw FFT data. Top: Surface plot from 300 FFT frames. Bottom: Beat frequencies overlaid on the raw data trace.

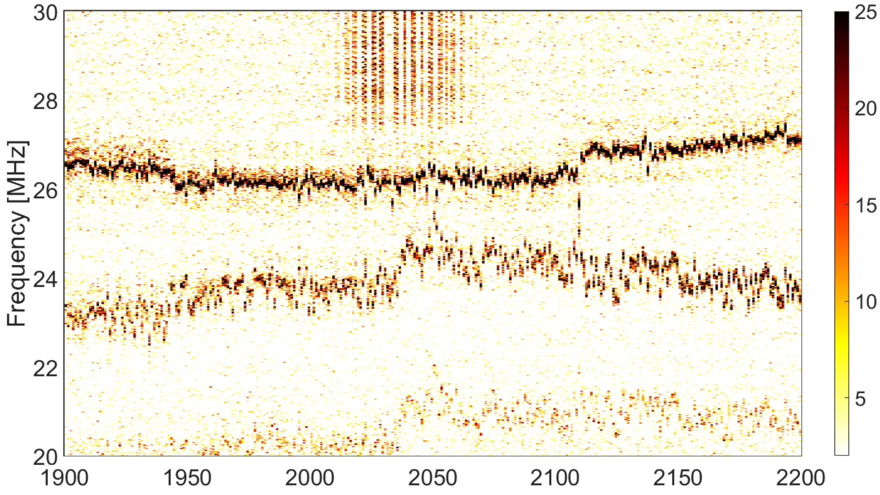

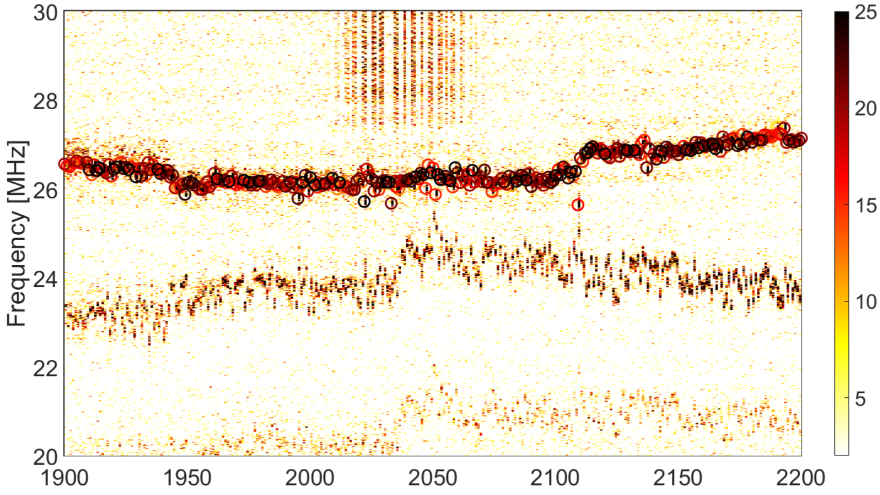

FIG. 2. Beatnote frequency extraction in a multimode lasing system. Top: Stacked FFT frames. Bottom: Corresponding beatnote signal detection.

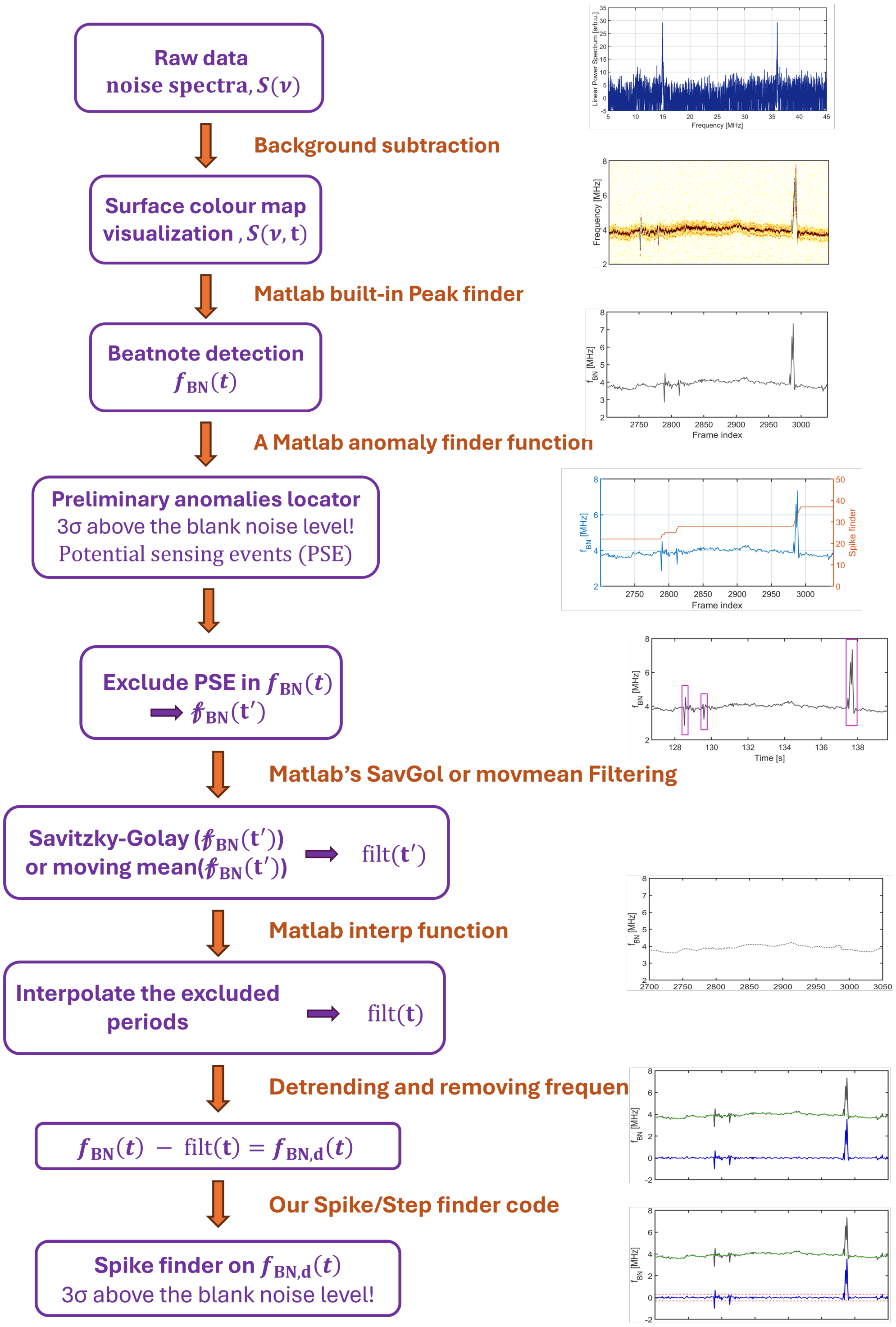

FIG. 3. Signal processing pipeline for identifying spikes. Steps include filtering, spike detection, and statistical analysis of spike features (time, amplitude, duration).

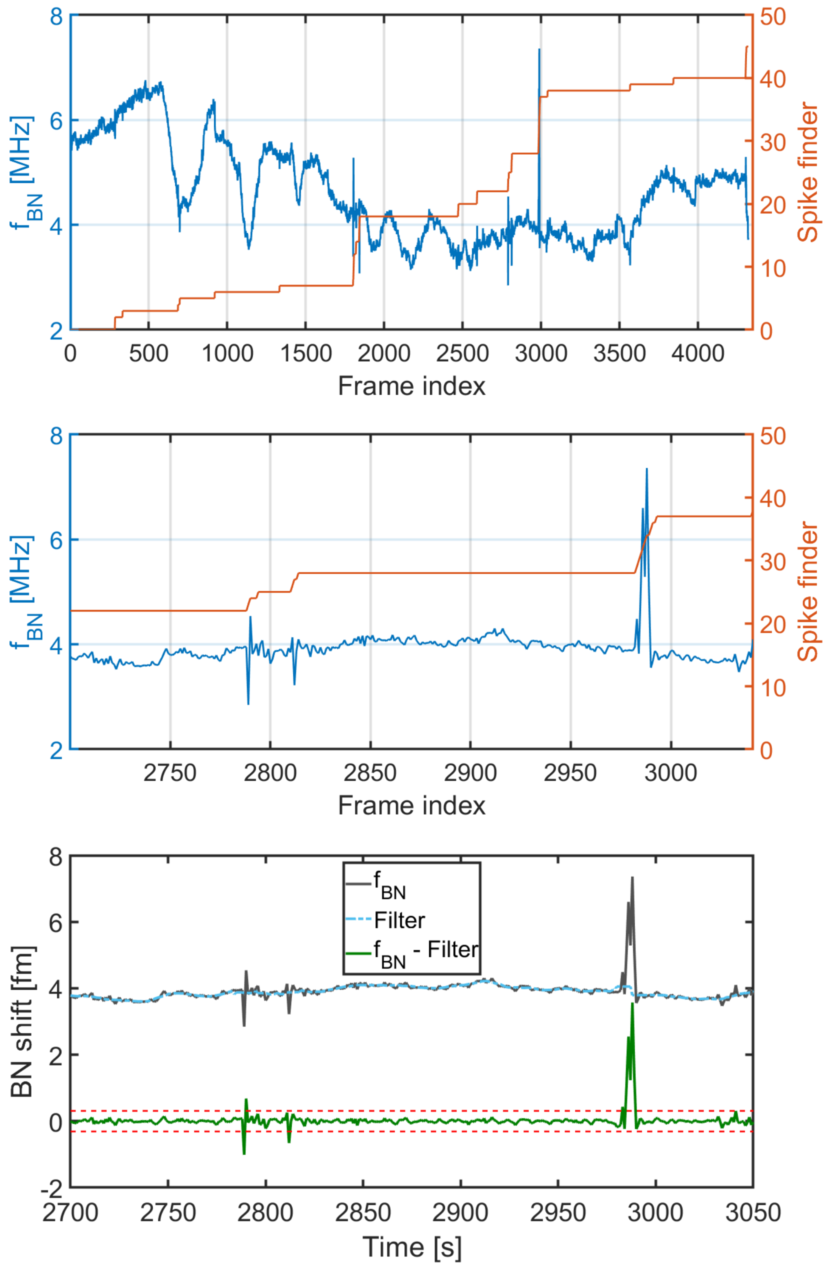

FIG. 4. Top: Raw beat frequency signal (blue) with spike detection overlay (orange). Middle: Zoomed-in view of a spike. Bottom: Detrending result using interpolation and filtering.

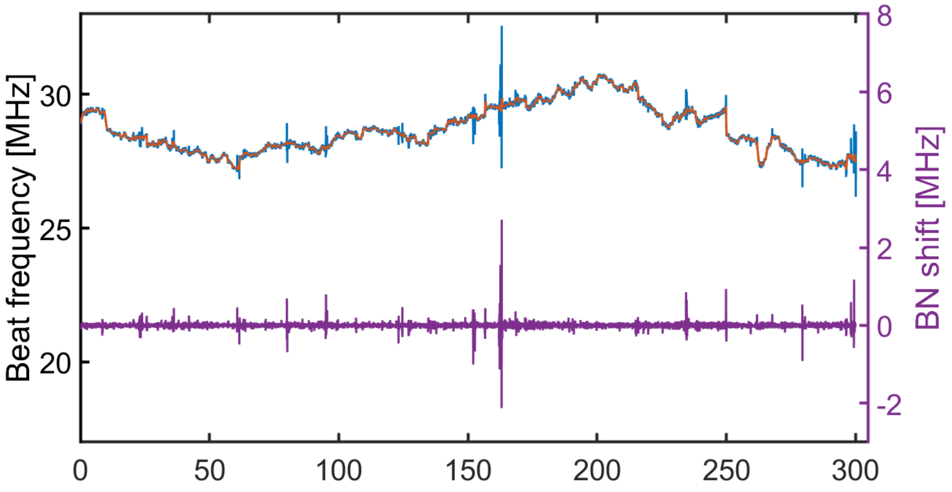

FIG. 5. Another example of detrending. Raw (blue), filtered (red), and final detrended signal (purple).

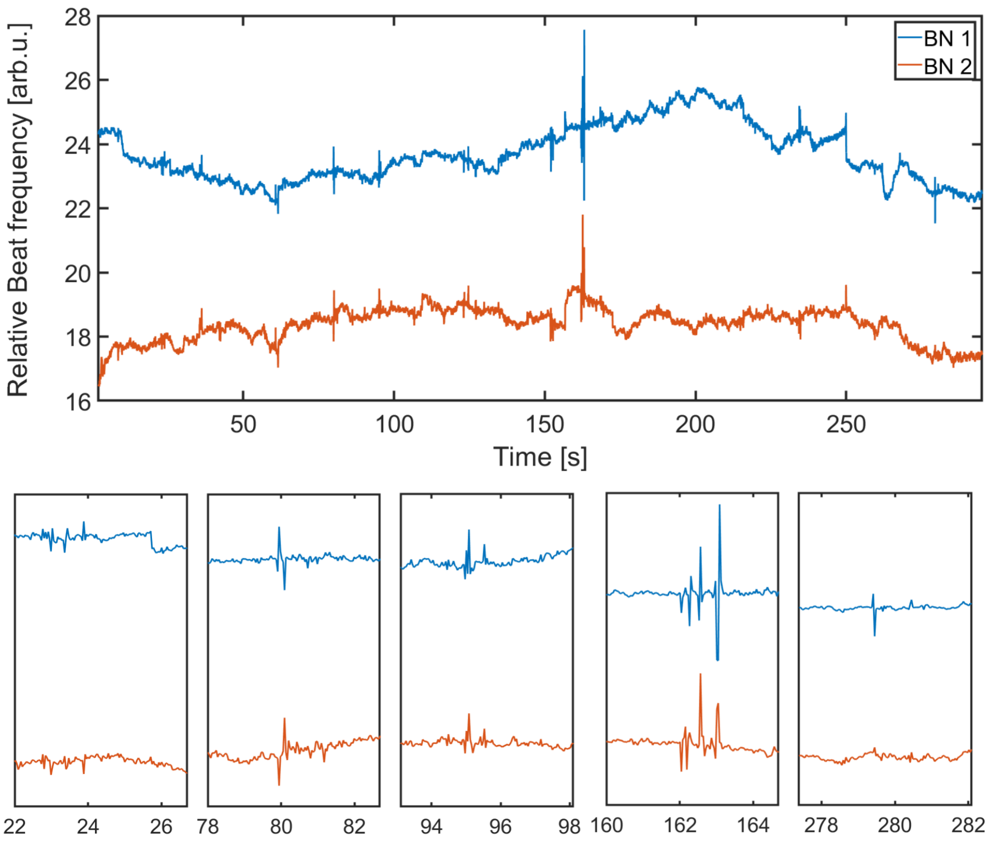

FIG. 6. Dual-mode beatnote analysis. Top: Time traces of two beatnote signals (slightly offset for clarity). Bottom: Zoomed-in comparison of events visible in one or both modes.

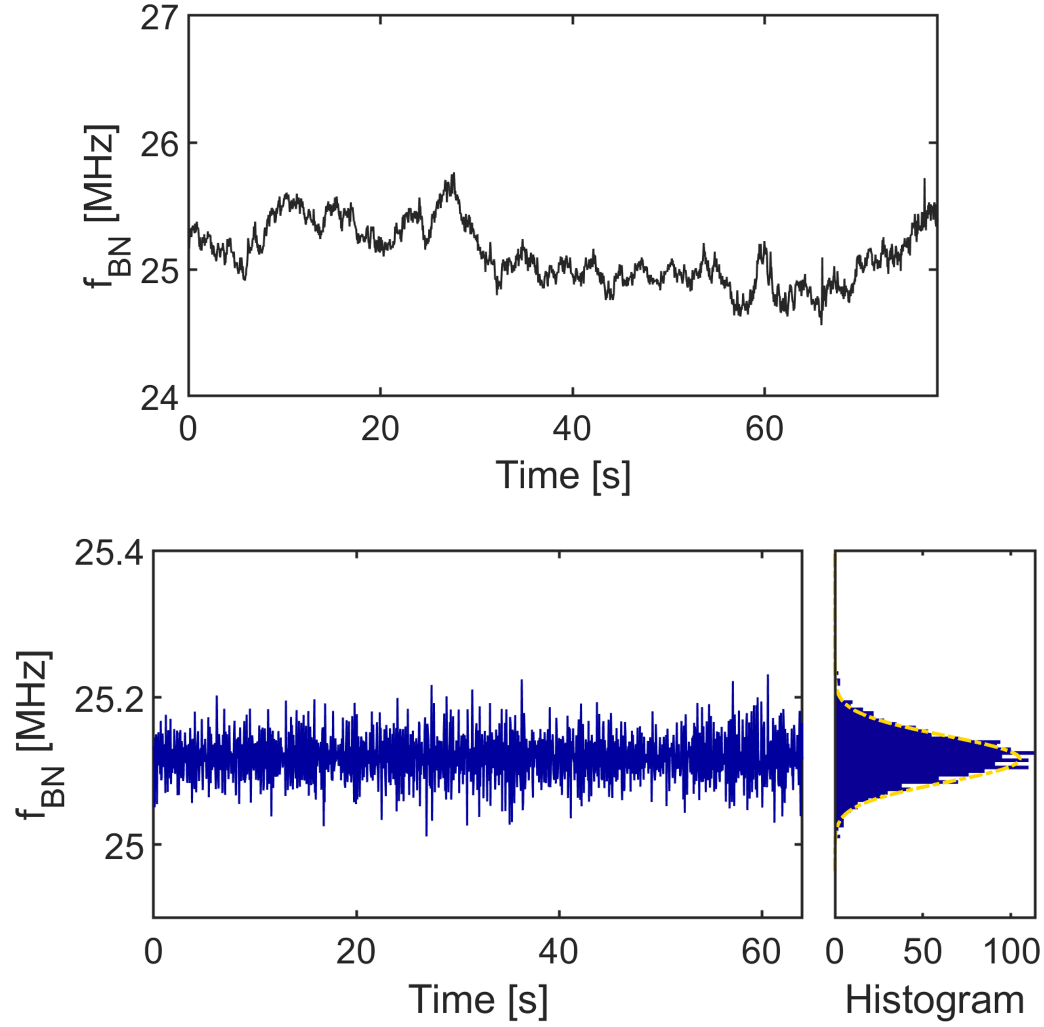

FIG. 7. The noise characterization shows a slow drift and variation in the beatnote frequency. This was acquired in a 5 mM aqueous solution of CsCL. The actual source of the slow fluctuations is unknown to us but we observed it is, at least partially, correlated to the detuning variations between the pump laser and the WGM and could be related to the phase matching of them. The local standard deviation in a span of 3 s (which is much longer than the sensing events) is measured at ~ 100 kHz, which at 1030 nm lasing wavelength corresponds to 0.4 fm bandwidth, and the maximum of the peak to peak fluctuations doesn't exceed 400 kHz. The right plot shows detrended background with its probability distribution function (PDF).

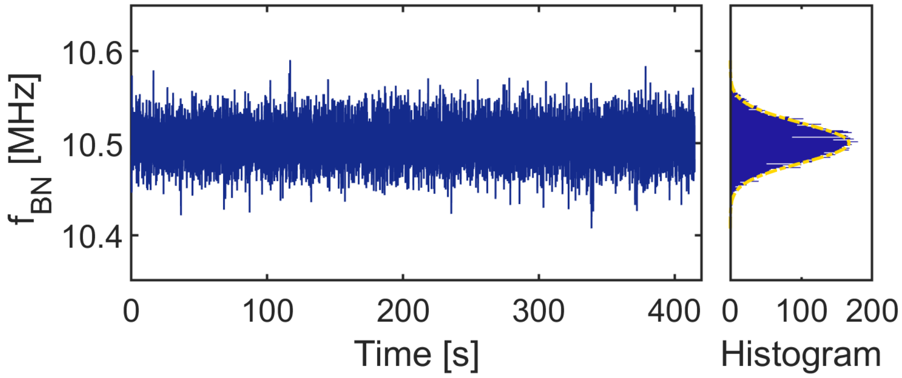

FIG. 8. Additional example of background noise PDF.

Appendix G: Noise level, detection limit, and PDF

As described in the previous section, after detrending the background data, we can extract the noise in our system (Figure 7, 8). A threshold level greater than three times standard deviation of the noise, 3σ, is used in the analysis to extract the spikes and steps events in our sensing data. Signals variation exceeding the threshold would be identified as potential sensing events. In this context, the noise is measured by monitoring the beatone frequency over time as the optical system of PE-WGMR is placed in the buffer. In the absence of the analyte, the slow drifts and fluctuations in the frequency of the beatnote characterize the noise in our system. This type of characterization is done prior to every single molecule or single atomic ion sensing experiments, which in this manuscript is referred to as the background characterization. In our experiments reported in this article, we measured a local standard deviation (over a span of $> 3\,\mathrm{s}$) $\sigma \sim 100\,\mathrm{kHz}$ or equivalently $0.4\,\mathrm{fm}$ bandwidth at the lasing wavelength.

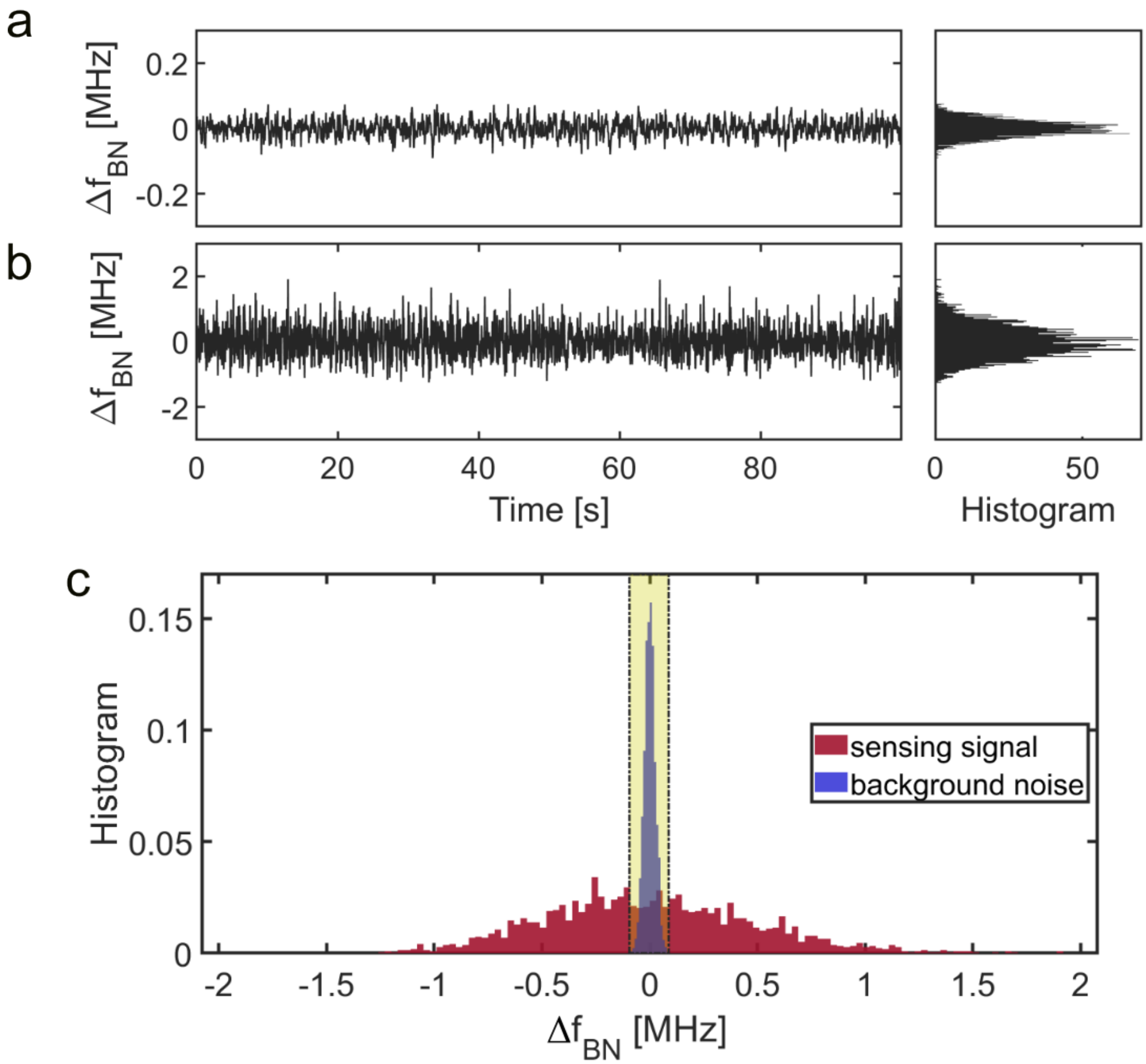

FIG. 9. **a**, Probability distribution function (PDF) of the beatnote frequency for a PE-WGM microlaser in a 5 mM CsCl buffer solution (background, no analyte). **b**, PDF of the beatnote frequency in the presence of $5\,\mu\mathrm{M}$ Cd^{2+} analyte. **c**, Normalized histograms comparing fluctuations in background and sensing signals.

The beatnote frequency trace was analyzed by computing its probability distribution function (PDF). Figure 9 compares the PDFs of the beatnote frequency for a PE-WGM microlaser in a 5 mM CsCl buffer solution (background)

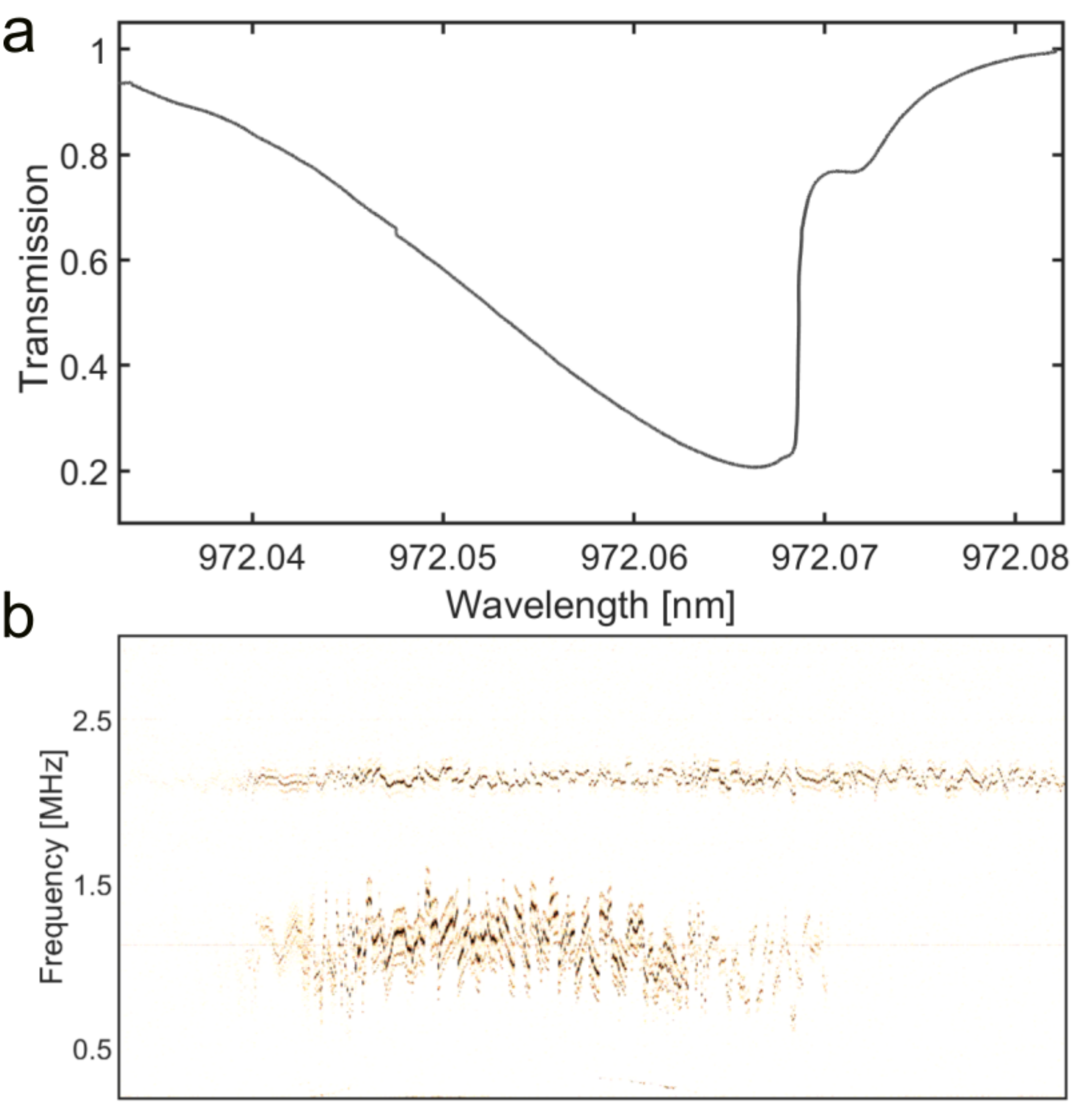

FIG. 10. **a**, Reconstructed transmission spectrum of a plasmon-enhanced WGM microlaser, obtained by linearly tuning the excitation laser wavelength. The asymmetric WGM profile indicates optothermal effects due to laser-induced heating and dissipation. **b**, Corresponding beatnote spectrum, showing the dependence of mode splitting and beatnote frequency on the excitation laser detuning from the WGM resonance.

and after introducing 5 μM Cd^{2+} analyte. This analysis highlights the shift in beatnote frequency distribution induced by Cd^{2+} interactions with the CTAB-capped gold nanorods.

Appendix H: Data Analysis and histograms

In this section we show all the relevant histograms (Fig. 11) for the detection of Zn^{2+} at different concentrations of 2.5, 10, 35 μM in 5 mM CsCl buffer. The beatnote analysis revealed the statistical properties of the sensing spikes amplitude, which are bidirectional e.g. up or down spikes. A dual Gaussian fit was applied to the histogram in a form of $y_{fit} = a_1 \exp\Big(-(\frac{x-b_1}{c_1})^2\Big) + a_2 \exp\Big(-(\frac{x-b_2}{c_2})^2\Big)$. Moreover distributions of the dwell time, τ, and the waiting time, ΔT, are obtained from the spike durations and time difference between two successive events. They fit well with exponential decay function as $\exp(-\tau/\tau_c)$ and $\exp(-\mathrm{r}_s\Delta T)$, owing to their Poisson statistics, which is due to the randomness of the single atoms movement in the medium. Table I shows the fit parameters for the histograms demonstrated in Fig. 11. The increase in the dwell time constant τ_c as a function of the ion concentration reflects the

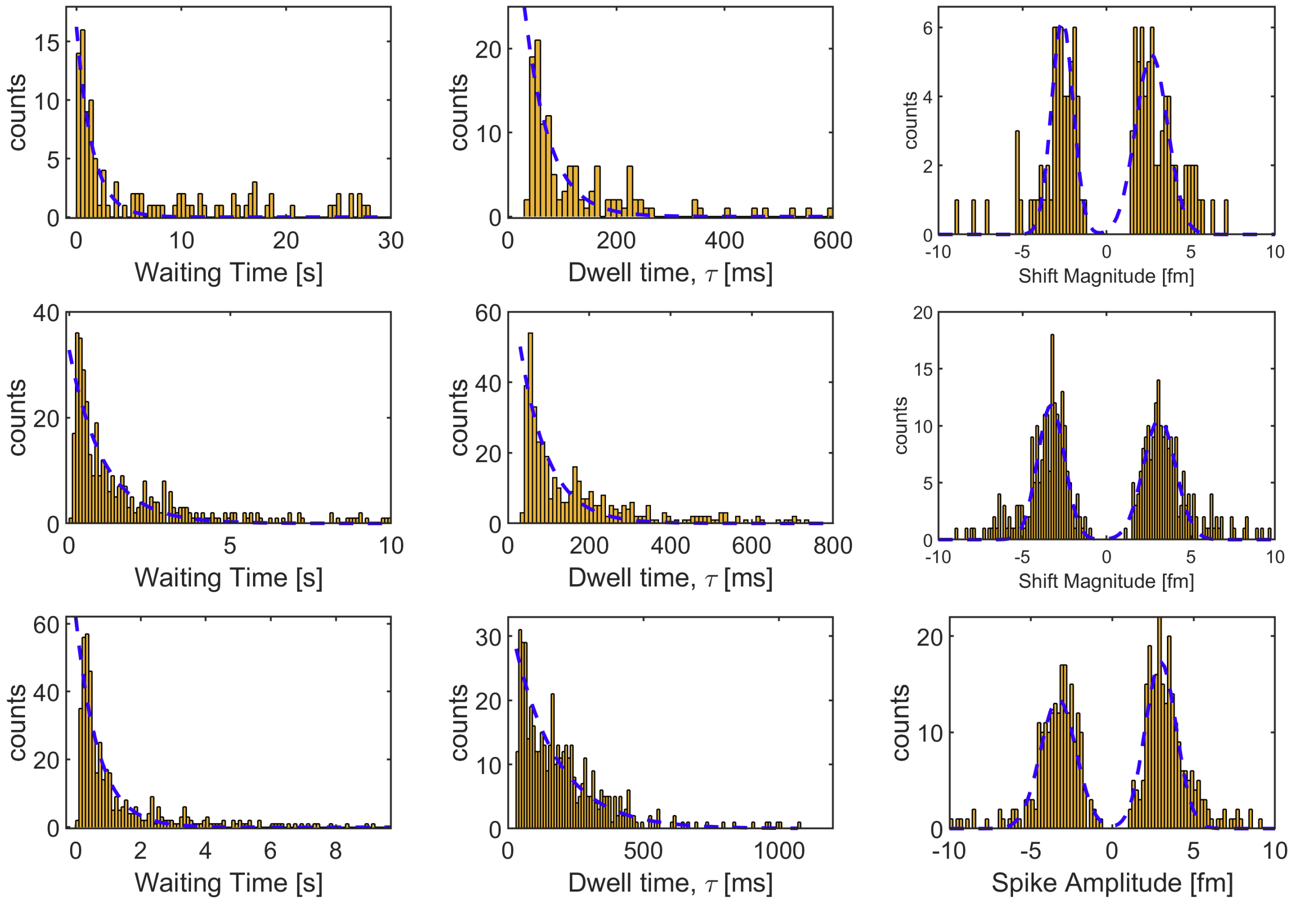

FIG. 11. Analysis and histograms for the zinc detection experiments for 2.5 μM (first row), 10 μM (second row), and 35 μM (third row).

fact that by increasing the ion concentration the charge screening at gold NR tips is enhanced, leading to prolonged interaction of the ion-NRs. It is known that by tuning the charge screening, the ion-NRs interaction tends toward more permanent binding [10].

Zn^{2+} Concentration	Spike amplitude 2^{ble} gaussian fit				Dwell time fit	Waiting time fit
C [μM]	b_1 [fm]	b_2 [fm]	c_1 [fm]	c_2 [fm]	τ_c [ms]	r_s [s^{-1}]
2.5	2.67 ± 0.07	-2.63 ± 0.05	1.35 ± 0.1	0.91 ± 0.07	$45.6 + 6.3 - 4.9$	0.66 ± 0.09
10	3.13 ± 0.05	-3.3 ± 0.04	1.35 ± 0.07	1.13 ± 0.06	$69.2 + 11.1 - 8.5$	0.95 ± 0.10
35	3.01 ± 0.03	-3.28 ± 0.03	1.44 ± 0.04	1.34 ± 0.04	$168.8 + 21.5 - 17.1$	1.45 ± 0.15

TABLE I. The fit parameters obtained in our spike characterization at different zinc concentration.

Appendix I: Subsequent backaction and treatment of oscillatory spikes

The term subsequent backaction refers to the secondary, counteracting frequency excursion that follows the initial ion-induced mode splitting. It arises from the interplay between two effects triggered by the adsorption event:

- The ion transiently shifts both lasing modes (most likely via reactive plasmonic response and detuning of narrow-

linewidth pump with respect to WGM giving photothermal response), changing the beat-note frequency.

- This shift briefly alters the intracavity power and the thermal locking condition, as it slightly detunes the pump laser-WGM. The self-thermal-locking feedback then pulls the system back toward the original detuning and meanwhile the ion walks away, producing an opposing frequency excursion of the lasing modes (and thus of the beatnote). These two processes of the thermal readjustment and analyte departing the sensor possess similar bandwidths which lead to a stronger counteraction and overshooting.

This back-and-forth dynamic often manifests as an oscillatory spike: an initial peak (or dip) caused by the ion, followed by a peak of opposite polarity driven by the thermal re-locking in combination with the effect of ion walking away (clearly visible in Fig. 2d and in traces in the Supporting Information). The phenomenon is intrinsic to thermally locked active WGM microlasers.

For every detected transient event—whether it shows a simple monopolar spike or a clear bipolar/oscillatory shape—we apply the following consistent procedure:

- The entire transient is treated as a single binding event.
- The largest-amplitude peak (regardless of whether it is the initial ion-induced peak or the subsequent backaction peak) is taken as the representative spike amplitude.
- The amplitude ratio is then computed from these two maximum values, and the sign of the ratio is preserved exactly as measured.

Appendix J: Sensing nanoparticles

We evaluated the WGM microlaser for nanoparticle sensing. In one experiment, a 100 μm resonator immersed in a 20 mM NaCl solution detected NaCl nanocrystals of varying sizes. Fig. 12 shows step-like shifts in the beatnote frequency, indicative of nanoparticle adsorption on the microlaser surface. The resonator, operating in multiple lasing modes, exhibited selective detection, with step events observed in specific modes (e.g., at frames 7800 and 7900) but absent in others, as revealed by the surface plot. Bottom images highlight nanoparticle attachment (marked by red circles) during the acquisition period. In a separate experiment, deposition of CTAB-capped gold NRs, identical to those in the main text, was monitored in a 24 mM HCl buffer, with binding events detected as beatnote frequency shifts (Fig. 13).

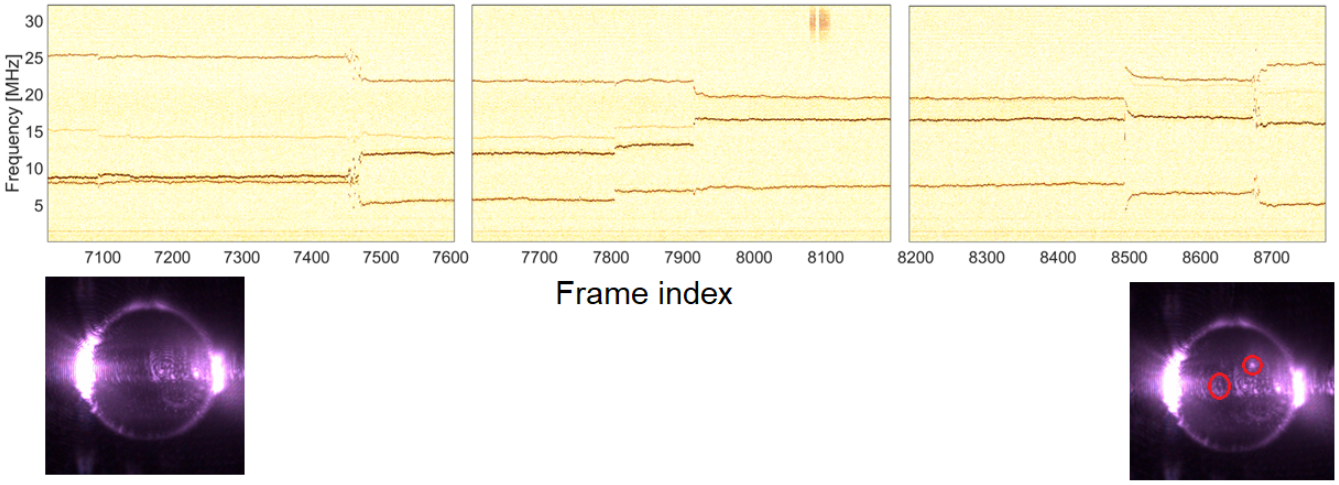

FIG. 12. Detection of sodium chloride crystal nanoparticles in water via step shifts in beatnote frequency. The scattering of the newly adsorbed nanoparticles are marked with a red circle on the microsphere image (bottom right) and can be compared with the image before the deposition.

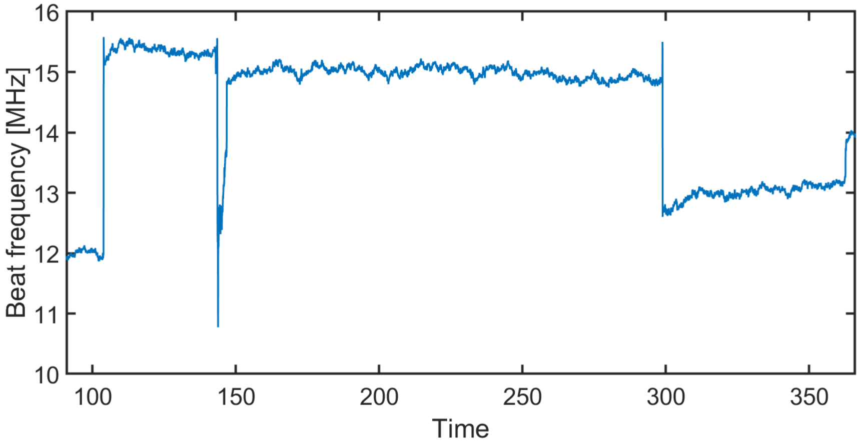

FIG. 13. Detecting AuNRs with length of 67 nm and diameter of 10 nm in a buffer of 24 mM HCl (pH=1.6).

Appendix K: sensing single molecules

In this experiment, 50 nm Gaba in water was used in order to test the single-molecule capability of our microlaser sensor, as reported in the main manuscript. In this experiment a 90 μm resonator with two lasing modes was used. The resulted beatnote signals appeared very close to each other (Fig. 14). However, the magnitude of them was different, makes it possible to track both beatnote simultaneously. As shown in Fig. 14, both steps and spikes were obseved. The shift in the beatnote signals, for some sensing events are in the same direction and sometimes in the opposite direction of each other.

Analyte	Chemical formula	Charge	Molecular weight (g/mol)	Polarizability ($\mathring{A}^3$)	step/spike amplitude (fm)
**Zwitterionic GABA**	$C_4H_9NO_2$	0	103.12	~ 9.5	4.1 ± 0.7
**Zinc ion (Zn^{2+})**	Zn	+2	65.38	2.8	3.7 ± 0.1
**Cadmium ion (Cd^{2+})**	Cd	+2	112.41	7.2	7.2 ± 0.1

TABLE II. Information about the analyte used in this research. The polarizability estimated for GABA in neutral form (zwitterionic in physiological conditions). Polarizability depends on molecular conformation and environment (e.g., gas phase vs. solution) [11]. Transition metals like zinc and cadmium have moderate polarizability due to their electron configuration [12]

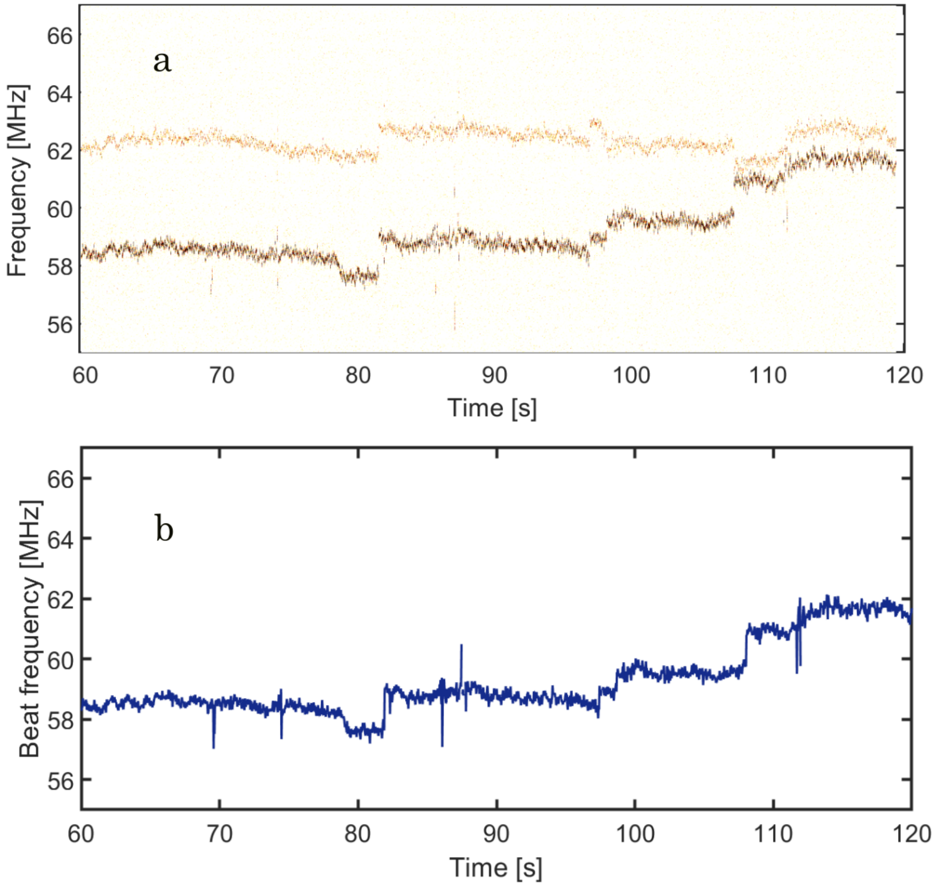

FIG. 14. Here we show the detection of 50 nM gamma-aminobutyric acid (Gaba) in water which clearly forms both transient binding and permanent binding. **a**, The average amplitude of the beatnote signal is about 23 and 12 dBm. **b**, The stronger beatnote signal was extracted and analyzed.

[1] L. Slooff, M. De Dood, A. Van Blaaderen, and A. Polman, Effects of heat treatment and concentration on the luminescence properties of erbium-doped silica sol–gel films, Journal of non-crystalline solids **296**, 158 (2001).
[2] Y. Huang, Y. Huang, P. Zhang, and C. Guo, Ultralow-threshold laser and blue shift cooperative luminescence in a yb3+ doped silica microsphere, AIP Advances **4** (2014).
[3] L. He, Ş. K. Özdemir, J. Zhu, and L. Yang, Ultrasensitive detection of mode splitting in active optical microcavities, Physical Review A—Atomic, Molecular, and Optical Physics **82**, 053810 (2010).
[4] C. Baes and R. E. Mesmer, The hydrolysis of cations 1976, Malabar, Fla.: RE Krieger (1986).
[5] A. K. Arunkumar, E. Zossimova, M. Walter, S. Pedireddy, J. Xavier, and F. Vollmer, Probing the single neurotransmitters with the wgm microcavity-hybridized plasmonic nanospiked antennas, arXiv e-prints arXiv:2507.10146 , arXiv (2025).
[6] S. Heiles, G. Berden, J. Oomens, and E. R. Williams, Competition between salt bridge and non-zwitterionic structures in deprotonated amino acid dimers, Physical Chemistry Chemical Physics **20**, 15641 (2018).
[7] M. L. Mayes and L. Perreault, Probing the nature of noncovalent interactions in dimers of linear tyrosine-based dipeptides, ACS omega **4**, 911 (2019).
[8] G. Puentes, Laser frequency offset locking scheme for high-field imaging of cold atoms, Applied Physics B **107**, 11 (2012).
[9] V. Li, F. Diorico, and O. Hosten, Laser frequency-offset locking at 10-hz-level instability using hybrid electronic filters, Physical Review Applied **17**, 054031 (2022).
[10] M. D. Baaske and F. Vollmer, Optical observation of single atomic ions interacting with plasmonic nanorods in aqueous solution, Nature Photonics **10**, 733 (2016).
[11] E. Zossimova, *Computational Modelling of Nanoscale Interactions for Nanophotonics and Biosensing* (University of Exeter, PhD thesis, 2024).
[12] R. D. Shannon, Dielectric polarizabilities of ions in oxides and fluorides, Journal of Applied physics **73**, 348 (1993).
